# Pediatric chronic myeloid leukemia with inv(3)(q21q26.2) and T lymphoblastic transformation: a case report

**DOI:** 10.1186/s40364-016-0069-0

**Published:** 2016-07-22

**Authors:** Margaret Lewen, Renee Gresh, Maria Queenan, Michele Paessler, Vinodh Pillai, Elizabeth Hexner, Dale Frank, Adam Bagg, Richard Aplenc, Emi Caywood, Gerald Wertheim

**Affiliations:** Department of Medicine, Boston Children’s Hospital, Boston, Massachusetts USA; Nemours Center for Cancer and Blood Disorders, Nemours/Alfred I. duPont Hospital for Children, Wilmington, Delaware USA; Department of Pathology and Laboratory Medicine, Nemours/Alfred I. duPont Hospital for Children, Wilmington, Delaware USA; Department of Pathology and Laboratory Medicine, Children’s Hospital of Philadelphia, Philadelphia, Pennsylvania USA; Department of Pathology and Laboratory Medicine, Perelman School of Medicine at the University of Pennsylvania, Philadelphia, Pennsylvania USA; Department of Medicine, Division of Oncology, Perelman School of Medicine at the University of Pennsylvania, Philadelphia, Pennsylvania USA; Division of Oncology, Children’s Hospital of Philadelphia, Philadelphia, Pennsylvania USA

**Keywords:** Chronic myeloid leukemia, Blast phase, Additional chromosomal abnormalities, MECOM, Case report

## Abstract

**Background:**

Chronic myeloid leukemia (CML) comprises ~3 % of pediatric leukemia. Although therapy with tyrosine kinase inhibitors (TKIs) is highly effective for CML, multiple factors have been identified as predictive of treatment failure. Chromosomal abnormalities involving the *MECOM* locus at 3q26 portend therapy resistant disease in adults, yet have never been described in pediatric patients and have not been associated with T lymphoblastic progression.

**Case presentation:**

We present a case of an 11-year-old boy with CML possessing the unique combination of T lymphoblastic transformation and a subclone harboring inv(3)(q21q26.2) at diagnosis. This is the first reported case of pediatric CML with inv(3)(q21q26.2) and the first case of T lymphoblastic progression associated with this karyotype. The patient was treated with single agent TKI therapy with robust initial response. Marrow histology at one month showed restoration of trilineage hematopoiesis and *BCR-ABL* RT-PCR at three months showed a 1.4 log reduction in transcript levels.

**Conclusions:**

The karyotypic abnormality of inv(3)(q21q26.2) in CML is not restricted to adult patients. Moreover, while chromosome 3 abnormalities are markers of TKI resistance in adults, our patient showed a robust early response to single agent TKI therapy. This finding suggests pediatric CML with inv(3)(q21q26.2) may have distinct features and more favorable treatment responses than those described in adults.

## Background

Chronic myeloid leukemia (CML) is a myeloproliferative neoplasm characterized morphologically by overproduction of maturing granulocytes and genetically by the *BCR-ABL1* fusion oncogene. CML constitutes 15–20 % of adult leukemia [1] yet is uncommon in children, comprising only 2–3 % of all pediatric leukemia [2]. The natural history of CML is either biphasic or triphasic, with progression from an indolent chronic phase (CP) to a terminal blast phase (BP), occasionally through an intermediate or accelerated phase (AP). Advanced disease is infrequent at diagnosis, with only 15 % of adult and 5 % of pediatric patients initially presenting with AP or BP [2, 3]. Morphologically, BP resembles acute leukemia and is not restricted to the myeloid lineage, indicating that very early hematopoietic progenitors harbor the *BCR-ABL1* translocation. Between 50–65 % of CML-BP shows myeloid differentiation, while lymphoid and undifferentiated phenotypes comprise 20–25 % and 15–25 %, respectively [4, 5]. The majority of lymphoid BP in CML is B lymphoblastic, while T lymphoblastic transformation is rare.

The hallmark karyotypic abnormality of CML is t(9;22)(q34;q11), yet complex translocations, such as t(6;9;22), are seen in 5–10 % of cases. The resulting BCR-ABL1 fusion protein is sensitive to imatinib and related tyrosine kinase inhibitors (TKIs). Use of these agents has vastly improved prognosis; however, a subset of patients progress to AP or BP despite adequate treatment, and prognosis for CML-BP remains poor [6].

Progression from CP to AP and BP is associated with acquisition of additional chromosomal abnormalities (ACAs). ACAs of trisomy 8, isochromosome 17q, and Philadelphia chromosome amplification, often referred to as “major-route” changes, serve as genetic markers of high-risk disease and are, therefore, sufficient for classifying CML-AP [5, 7]. Less frequent “minor-route” ACAs are more varied and have poorly described treatment implications. One notable exception is abnormalities of 3q26.2 resulting in overexpression of the *MECOM* locus [8, 9]. Increased expression of *MECOM*, an oncogenic transcription factor involved in hematopoietic stem cell renewal and differentiation, is associated with resistance to TKIs and progression to myeloid CML-BP [10–13]. Recently, rearrangements involving the 3q26.2 locus have been shown to be an independent predictor of inferior outcomes in adults with CML [14]. As such, 3q26.2 alterations, found either directly by genetic assays or indirectly by determination of MECOM expression levels, may be markers for subclassification of patients with CML. Of note, 3q26.2 locus abnormalities have not been reported in pediatric CML and thus are of uncertain significance in this population.

We report a case of pediatric CML with the unique combination of T lymphoblastic progression and a subclone harboring inv(3)(q21q26.2) at diagnosis. This is the first reported case of CML in which inv(3)(q21q26.2) occurs simultaneously with T lymphoblastic progression, as well as the first reported instance of inv(3)(q21q26.2) in pediatric CML. Notably, immunohistochemical staining (IHC) of the bone marrow using a novel antibody against MECOM demonstrated the T lymphoblastic population to be independent of the subpopulation with inv(3)(q21q26.2). Despite these adverse features, the patient initially responded well to TKI monotherapy. We discuss the diagnostic, prognostic, and therapeutic implications for this patient and for patients with CML with inv(3)(q21q26.2) in general.

## Case presentation

The patient is an 11-year-old male with attention deficit hyperactivity disorder (ADHD) who presented with an unintentional 20 lb. weight loss that was initially attributed to ADHD medication. Exam was notable for cervical lymphadenopathy. Laboratory workup showed leukocytosis with left-shifted granulocytes (WBC 210,000 cells/μL; 65 % neutrophils, 6 % bands, 5 % lymphocytes, 3 % monocytes, 2 % eosinophils, 3 % metamyelocytes, 13 % myelocytes, 2 % promyelocytes, 1 % blasts), normocytic anemia (Hgb 10.8 g/dL, MCV 92 fL), elevated LDH (1,858 U/L), and elevated uric acid (7.0 mg/dL). Abdominal CT scan showed splenomegaly.

**Fig. 1:**
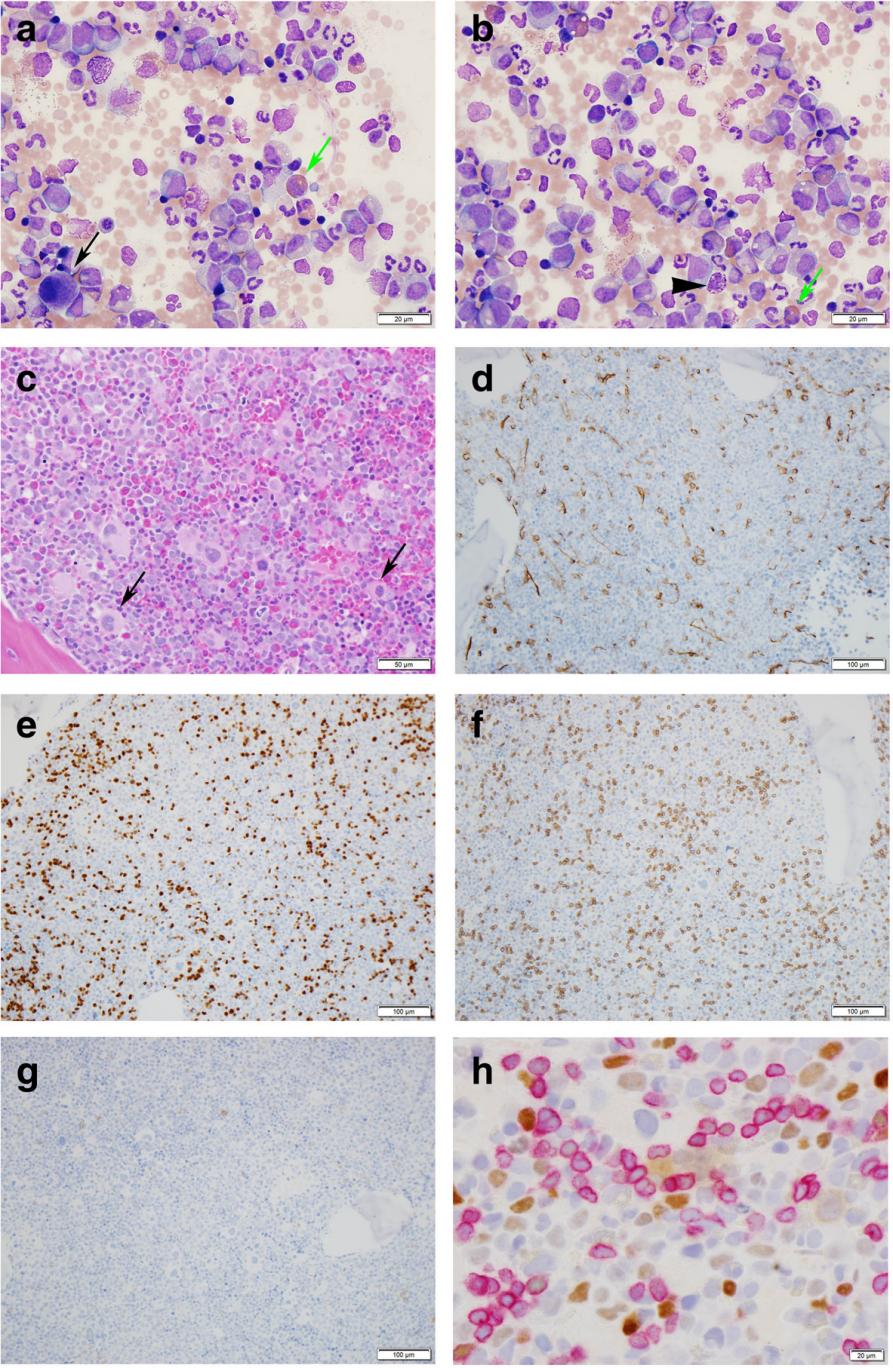
Morphologic and immunohistochemical evaluation of diagnostic bone marrow aspirate and core biopsy. Diagnostic bone marrow aspirate (**a** and **b**, Wright-Giemsa, 600x) and biopsy (**c**, **h** & **e**, 200x) showing myeloid elements at all stages of maturation. The aspirate shows that blasts are not markedly increased and cells with cytology consistent with lymphoblasts are not readily apparent. Eosinophils (green arrows), basophils (arrowhead) and dwarf megakaryocytes (black arrows) are identified. Immunohistochemical staining of the bone marrow biopsy (100x) shows an expansion of CD34(subset) + TDT+ blasts (**d** and **e**, respectively) that form clusters. An expansion of CD3+ T-cells (**f**) without an expansion of CD19+ B-cells (**g**) is seen. In panel **h**, double staining of the biopsy (400x) shows that MECOM expressing cells (brown nuclear stain) are distinct from CD3+ T-cells (red membranous stain)

A bone marrow study revealed hypercellular marrow (>95 %) with increased myeloid cells at all stages of maturation, increased eosinophils, scattered basophils, few erythroid progenitors, so-called dwarf megakaryocytes, and sea-blue histiocytes (Fig. 1a–d). Blasts were <5 % morphologically. Flow cytometry identified a population of atypical CD3+ MPO- TDT+ T lymphoblasts (9 % of total events). IHC of the bone marrow core biopsy confirmed these latter findings, showing individual and clusters of CD3+ TDT+ T lymphoblasts comprising ~10 % of overall cellularity and 20–30 % of cells in restricted areas (Fig. 1d–g).

RT-PCR from peripheral blood was positive for the *BCR-ABL1* p210 transcript. Cytogenetic analysis of the bone marrow showed 46,XY,t(6;9;22)(p22;q34;q11.2)[9]/46,sl,inv(3)(q21q26.2)[11], confirming the presence of a variant three-way translocation generating the *BCR-ABL1* fusion. The presence of a subclone (11 of 20 cells analyzed) with inv(3)(q21q26.2) suggested disease progression. Following identification of this inversion and validation of a novel MECOM antibody, combined IHC for MECOM and CD3 was performed on the bone marrow core biopsy. Interestingly, the CD3+ population and the MECOM-overexpressing population were non-overlapping (Fig. 1h), indicating the T lymphoblastic transformation was independent of the acquisition of inv(3)(q21q26.2).

Based on these findings, a diagnosis of CML with T lymphoblastic transformation was rendered. The patient was started on hydroxyurea and allopurinol, followed by single-agent treatment with imatinib (500 mg daily). Repeat bone marrow studies on day 25 of treatment showed restoration of trilineage hematopoiesis and normal cellular morphology, with abnormal T lymphoblasts comprising 1 % of total cellularity by flow cytometry. Subsequent to this study, he was transitioned from imatinib to dasatinib (100 mg daily) due to the development of oral ulcers, and continued to improve clinically on TKI alone. A third bone marrow biopsy and aspirate at day 54 showed 0.02 % T lymphoblasts. Peripheral blood quantitative RT-PCR analysis at three months showed a 1.4 log reduction of *BCR-ABL1* transcripts (4.3 % IS units) (Table 1). Despite this response to TKI monotherapy, a matched unrelated stem cell donor was identified and transplantation is scheduled given the high-risk features of his disease.

**Table 1:** Clinical course

Day of TKI treatment	Clinical event	qPCR BCR/ABL, Blood (IS units)	qPCR BCR/ABL, Marrow (IS units)	T lymphoblasts, Marrow
−1	CML diagnosis confirmed	44 %		9 %
1	Started imatinib 500 mg daily			
22		58 %		
25			65 %	1 %
29		69 %		
34		50 %		
41		67 %		
49	Started dasatinib 100 mg daily	56 %		
54		52 %	52 %	0.02 %
64		28 %		
68		22 %		
92		4.3 %		

Timeline of clinical events, *BCR-ABL1* transcript levels detected by qPCR in the blood and bone marrow, and percentage of T lymphoblasts in the bone marrow as measured by flow cytometry. Timeline is reported relative to day 1 of treatment with imatinib. *TKI* tyrosine kinase inhibitor, *qPCR* quantitative polymerase chain reaction, *IS* international standard units

## Conclusions

We describe a case of pediatric CML with variant translocation t(6;9;22)(p22;q34q11.2) and two identifiable subclonal populations at presentation, one of which harbors inv(3)(q21q26.2) while the other is comprised of abnormal T lymphoblasts. CML is rare in the pediatric population, and only 5 % of patients have evidence of advanced disease at presentation [2]. Given the T lymphoblasts identified at diagnosis, a primary lymphoblastic process was considered; however, many of the features of this case favor a diagnosis of CML with T lymphoblastic progression, including: splenomegaly; peripheral blood with left-shifted granulocytosis and a marked increase in myelocytes; myeloid hyperplasia, dwarf megakaryocytes, and sea-blue histiocytes in the marrow; and t(6;9;22) producing the p210 *BCR-ABL1* transcript. Although the possibility of two independent processes (CML and T lymphoblastic lymphoma) was not formally disproven, a single disease entity (CML with progression) is more likely.

T lymphoblastic progression of CML is particularly rare [5]. Our patient did not meet formal WHO morphologic or genetic criteria for AP or BP [7]; however, recent studies in adult patients suggested flow cytometric detection of abnormal lymphoid populations at diagnosis to be highly predictive of rapid progression despite adequate treatment with TKIs [15]. Surprisingly, an expansion of lymphoblasts was not identified in the marrow aspirate smears, which is likely due to the non-uniform distribution of the lymphoblasts in the bone marrow. This finding indicates that extensive immunophenotypic and genetic evaluation of the marrow should be undertaken to rule out AP or BP even if the bone marrow aspirate smear demonstrate CP only.

Given the coincident T lymphoblastic transformation and inv(3)(q21q26.2), we performed IHC for both MECOM and CD3 and showed that the T lymphoblasts likely did not harbor the chromosome 3 inversion. The finding that these populations were independent was not altogether unexpected, as most cases of CML with inv(3)(q21q26.2) are associated with thrombocytosis, thrombosis, and dysmegakaryopoiesis rather than T lymphoblastic transformation [16]. Indeed, the most recent and comprehensive analysis of CML with chromosome 3 alterations [14] contained no cases with T lymphoblastic transformation.

MECOM is a zinc finger transcription factor whose overexpression promotes leukemogenesis via several mechanisms, including apoptotic resistance through direct transcriptional modulation [17] and epigenetic changes via promoter DNA methylation [18]. *MECOM* has been extensively studied in acute myeloid leukemia and myelodysplastic syndromes, in which increased expression is associated with poor prognosis [7, 19–21]. High levels of *MECOM* have also been detected in CML-BP [10] as well as a subset of patients with CML-CP with resistance to TKIs [14, 22]. Currently, the customary method for detecting *MECOM* overexpression is restricted to RT-PCR from tumor mRNA and is not standard practice in the clinical setting. Our detection of MECOM protein expression in the bone marrow core biopsy suggests that IHC may be a useful clinical method of evaluating MECOM expression and may potentially serve as a marker for high-risk disease.

ACAs are common at diagnosis in CML in any phase of disease, and “major-route” cytogenetic changes such as trisomy 8, isochromosome 17q, and amplification of the Philadelphia chromosome suggest both clonal evolution and adverse prognosis. While *MECOM* abnormalities are not formal “major-route” alterations, recent analysis identified abnormalities involving 3q26.2 in roughly 3 % of adult patients with CML and found them to be at particular risk for disease progression with near uniform unresponsiveness to TKIs [14]. In this study, none of the patients with inv(3)(q21q26.2) or t(3;3;)(q21;q26.2) showed sustained major molecular response (MMR) or complete cytogenetic response (CCyR). This finding was consistent with earlier studies in animal models demonstrating TKI resistance in Evi1-overexpressing cells in both CML-CP and CML-BP cells [12]. Additionally, studies in adult CML have found *MECOM* rearrangements to be associated with evolution of TKI resistance and progression to myeloid blast crisis despite adequate therapy [11, 13]. Given these associations, genetic detections of 3q26.2 abnormalities or immunohistochemical detection of MECOM overexpression in CML are potentially valuable markers for high-risk disease.

Based on the adult literature of CML with 3q26.2 rearrangements, we were unsure if our patient would respond to TKI monotherapy; however, after three months of treatment, *BCR-ABL1* transcript level had fallen to 4.3 % (IS). Multiple studies of adult (and a few adolescent) patients with CML receiving TKI therapy have found early molecular response (EMR), defined as *BCR-ABL1* transcript levels ≤10 % (IS) at 3 months, to be associated with significantly higher rates of overall survival, event- and progression-free survival, CCyR and MMR [23–25]: in fact, *BCR-ABL1* transcript level >10 % (IS) at three months was found to be the strongest predictor of poor clinical outcome [25]. Of note, the association between EMR and improved outcomes held true for patients who required transition to second-generation TKIs within the first three months due to imatinib failure or intolerance [24]. Our patient’s robust response to TKI monotherapy at three months suggests that clinical and biologic features of CML with inv(3)(q21q26.2) may be distinct in adult and pediatric patients.

Since the introduction of TKIs, hematopoietic stem cell transplant (HSCT) in CML has been largely regarded as salvage therapy for refractory disease. However, a recent large-scale prospective randomized comparison of TKIs and early HSCT found HSCT improved both long-term survival and sustained molecular remission in patients with high-risk disease and low transplant risk [26]. Given these results, combined with the lack of data in pediatric CML with inv(3)(q21q26.2), the high risk of TKI resistance in adult patients with this genetic abnormality, and the concomitant T lymphoblastic progression, we considered allogeneic stem cell transplant to be the best treatment option.

## Abbreviations

ACA, additional chromosomal abnormality; CCyR, complete cytogenetic response; CML, chronic myeloid leukemia; CML-AP, accelerated phase CML; CML-BP, blast phase CML; CML-CP, chronic phase CML; EMR, early molecular response; EVI-1, ecotropic virus integration site 1; HSCT, hematopoietic stem cell transplant; IHC, immunohistochemistry; IS, international standard units; MECOM, MDS1 and EVI1 complex locus; MMR, major molecular response; TKI, tyrosine kinase inhibitor
